# Module-based functional pathway enrichment analysis of a protein-protein interaction network to study the effects of intestinal microbiota depletion in mice

**DOI:** 10.3892/mmr.2014.2137

**Published:** 2014-04-09

**Authors:** ZHEN-YI JIA, YANG XIA, DANIAN TONG, JING YAO, HONG-QI CHEN, JUN YANG

**Affiliations:** Department of General Surgery, Shanghai Jiao Tong University Affiliated Sixth People’s Hospital, Shanghai 200233, P.R. China

**Keywords:** intestinal microbiota depletion, protein-protein interaction network, modules, pathway enrichment analysis

## Abstract

Complex communities of microorganisms play important roles in human health, and alterations in the intestinal microbiota may induce intestinal inflammation and numerous diseases. The purpose of this study was to identify the key genes and processes affected by depletion of the intestinal microbiota in a murine model. The Affymetrix microarray dataset GSE22648 was downloaded from the Gene Expression Omnibus database, and differentially expressed genes (DEGs) were identified using the limma package in R. A protein-protein interaction (PPI) network was constructed for the DEGs using the Cytoscape software, and the network was divided into several modules using the MCODE plugin. Furthermore, the modules were functionally annotated using the PiNGO plugin, and DEG-related pathways were retrieved and analyzed using the GenMAPP software. A total of 53 DEGs were identified, of which 26 were upregulated and 27 were downregulated. The PPI network of these DEGs comprised 3 modules. The most significant module-related DEGs were the cytochrome P450 (CYP) 4B1 isozyme gene (*CYP4B1*) in module 1, *CYP4F14* in module 2 and the tachykinin precursor 1 gene (*TAC1*) in module 3. The majority of enriched pathways of module 1 and 2 were oxidation reduction pathways (metabolism of xenobiotics by CYPs) and lipid metabolism-related pathways, including linoleic acid and arachidonic acid metabolism. The neuropeptide signaling pathway was the most significantly enriched functional pathway of module 3. In conclusion, our findings strongly suggest that intestinal microbiota depletion affects cellular metabolism and oxidation reduction pathways. In addition, this is the first time, to the best of our knowledge, that the neuropeptide signaling pathway is reported to be affected by intestinal microbiota depletion in mice. The present study provides a list of candidate genes and processes related to the interaction of microbiota with the intestinal tract.

## Introduction

There are >1,000 species of bacteria in the intestinal tract, known as intestinal microbiota. The genomes of these species encode >100-fold unique genes compared to the human genome (1). The intestinal microbiota is dominated by five bacterial phyla (Firmicutes, Bacteroidetes, Actinobacteria, Proteobacteria and Verrucomicrobia) and one Archaea (Euryarchaeota) (2). These complex communities of microorganisms play an important role in metabolic, nutritional, physiological and immunological processes in the human body (3). Molecular characterization of the intestinal microbiota by phylogenetic approaches has received considerable attention in recent years and revealed a remarkable compositional stability and resilience in adult life, even after pervasive treatments with antibiotics (4). Species of the genera *Bifidobacterium* and *Lactobacillus* are particularly present in the colon of healthy individuals, and they are generally regarded as desirable, owing to the reduction of the neutral pH to a more acidic pH that they cause (5). Changes in microbial community composition are closely associated with various diseases, such as allergic disease (6), colorectal cancer (7) and intestinal inflammatory disease (8).

Our understanding of intestinal microbiota and their importance for the human physiology has increased, owing to international research initiatives such as the MetaHIT project (1) and the Human Microbiome Project (9). However, the development of simple protocols for the manipulation of intestinal microbiota in experimental animal models is still needed. Recently, a study focusing on the effects of intestinal microbiota depletion on the gut mucosa and epithelial gene expression was performed; depletion of the intestinal microbiota was achieved in mice by administering broad-spectrum antibiotics in drinking water (10). The study reported that antibiotic treatment significantly reduced the expression of antimicrobial factors to a level similar to that of germ-free mice, and altered the expression of a total of 517 genes in the colonic epithelium. The expression of genes involved in the cell cycle was significantly altered, concomitant with reduced epithelial proliferative activity *in situ*, as assessed by Ki-67 expression, which suggested that commensal microbiota drives cellular proliferation in the colonic epithelium (10). Metabolites produced by the gut microbiota community from processes such as oxidation reduction and lipid metabolism have been reported to considerably affect intestinal functions (1).

The present study used a previously released microarray dataset (10) to assess the effects of intestinal microbiota depletion in mice, by focusing on the gene expression profiles of colonic intestinal epithelial cells in the presence and absence of intestinal microbiota. These profiles were analyzed using a series of bioinformatic methods, including protein-protein interaction (PPI) network construction, module functional annotation and pathway enrichment analyses. Further research on the mechanisms identified here as affected by the intestinal microbiota depletion is planned for a future study.

## Materials and methods

### Affymetrix microarray analysis

The raw data and the probe annotation files from the gene expression profiling dataset GSE22648 (10; accession no. GDS3921) were downloaded from the Gene Expression Omnibus database (the National Center of Biotechnology Information; http://www.ncbi.nlm.nih.gov/geo/query/acc.cgi?acc=GSE22648). These data were obtained on a GPL6887 platform using MouseWG-6 v2.0 expression beadchips (Illumina, Inc., San Diego, CA, USA). Data from a total of 11 chips were analyzed, corresponding to colonic intestinal epithelial cell gene expression profiles of 5 replicates from mice with depleted intestinal microbiota and 6 replicates from control mice that were not treated with antibiotics (germ-free).

### Identification and clustering analysis of differentially expressed genes (DEGs)

The raw data were preprocessed using the Affy package in R (11). Differential expression analysis between the 5 intestinal microbiota-depleted and the 6 control samples was performed using limma, a linear regression model software package available in R (12), and multiple testing correction was performed using a Bayesian method (13). The DEGs between intestinal microbiota-depleted and control samples were defined as these genes showing a |log fold change (FC)| >1 and a false discovery rate (FDR) <0.05. To visualize the expression profiles of DEGs and all genes, unsupervised hierarchical clustering analysis was performed (14).

### PPI network construction

The search tool for the retrieval of interacting genes (STRING) (15) database was used to retrieve the predicted interactions for the identified DEGs; the version 9.0 of STRING covers >1,100 completely sequenced species. All associations available in STRING are provided with a probabilistic confidence score, which was derived by separately benchmarking groups of associations against the a manually curated functional classification scheme (15). Each score represents a rough estimate of how likely a given association describes a functional linkage between two gene products. The DEGs with a confidence score >0.8 were selected to construct the PPI network, using the open-source Cytoscape software (16). Cytoscape (http://cytoscape.org/) allows visualizing complex networks and integrating these networks to any type of attribute data.

### Functional analysis of modules from the PPI network

The MCODE (17) plugin in Cytoscape was used to further divide the PPI into modules, using a cutoff value for the connectivity degree of nodes (proteins in the network) >2. Gene Ontology (GO) functional annotation and enrichment analysis of genes in the resulting modules was performed using the PiNGO plugin in Cytoscape (18) with a threshold P<0.05 based on a hypergeometric test.

### Pathway analysis

Information on the biological pathways in which the module-related DEGs are involved was retrieved from the Kyoto Encyclopedia of Genes and Genomes pathways database (http://www.genome.jp/kegg/pathway.html) (19,20). Visualization of these pathways and enrichment analysis was performed using the GenMAPP software (19). GenMAPP is a powerful tool for graphically viewing microarray data in the context of pathway analysis in an intuitive manner for biologists, and it was previously used in the analysis of microarray data related to allergic disease (21). P<0.05 was set as the threshold used for enrichment analysis of KEGG pathways.

## Results

### Identification of DEGs

**Figure 1:**
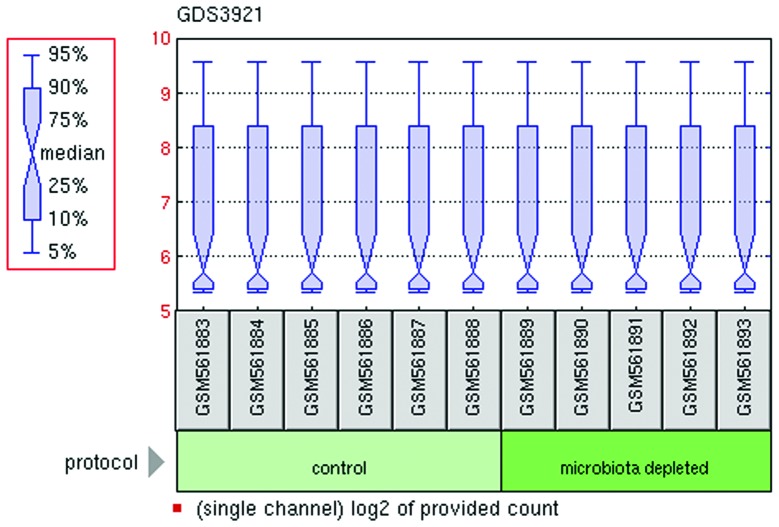
Boxplot of normalized expression values for the dataset (accession no. GDS3921). The data for the six control samples are presented on the left, and for the five samples with microbiota depletion on the right. The dotted line in the middle of each box represents the median of each sample, and its distribution among samples indicates the level of normalization of the data, with a nearly straight line revealing a fair normalization level.

The normalized expression values following preprocessing of the raw data are shown in Fig. 1. Differential expression analysis on these values using FDR<0.05 and |log FC|>1 as cutoff criteria identified a total of 53 genes differentially expressed between depleted intestinal microbiota and control mice. Among these DEGs, 26 were upregulated and 27 were downregulated upon microbiota depletion.

### Clustering analysis of DEGs

**Figure 2:**
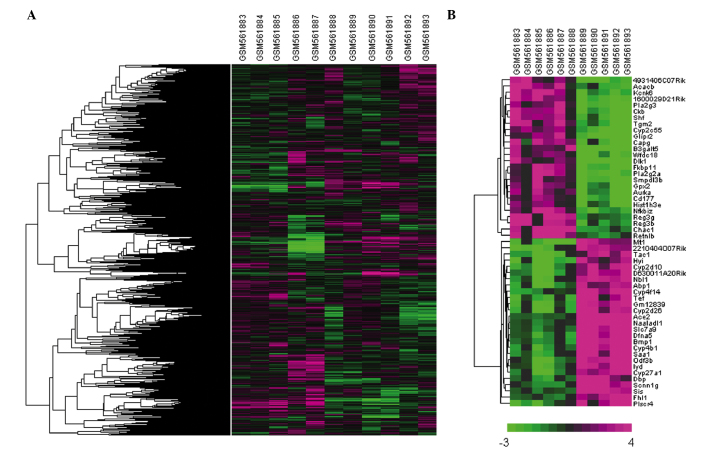
Clustering analysis of gene expression values of (A) all genes and (B) of differentially expressed genes. The change of color from green to red represents the change in |log FC| from low to high. FC, fold change.

Hierarchical clustering analysis was performed on the expression values of all genes and of the 53 DEGs. Clearly distinct expression patterns were observed between the microbiota-depleted and the control mice in both the total gene and DEG clustering analysis (Fig. 2).

### PPI analysis and module functional annotation

**Figure 3:**
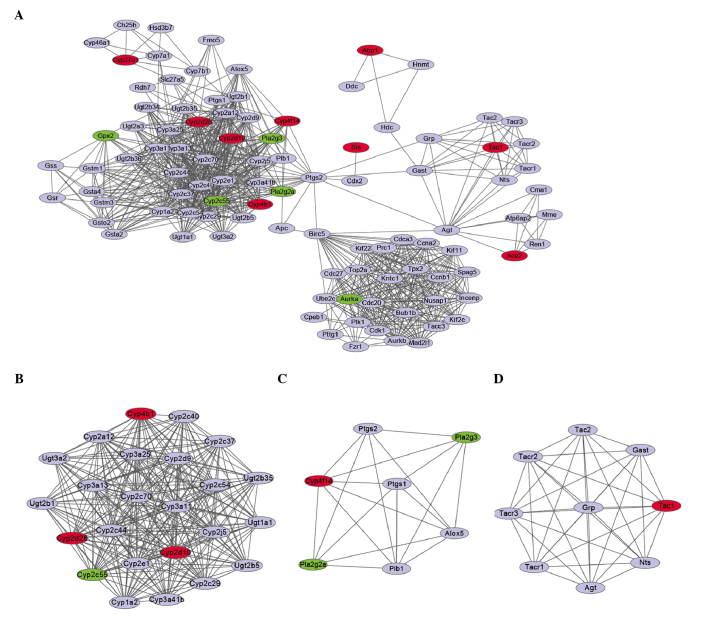
Primary protein-protein interaction (PPI) network and selected modules. (A) PPI network for products of differentially expressed genes (DEGs). (B–D) Modules including significant DEGs (confidence score >0.8). Red- and green-color nodes represent products of up- and downregulated DEGs, respectively. Purple nodes denote products of genes predicted to interact with the DEGs.

The PPI network was constructed (Fig. 3A) based on the predicted interactions of 14 DEGs showing a confidence score >0.8. Using the MCODE plugin in Cytoscape, the PPI network was divided into three modules (Fig. 3B–D). Modules 1, 2 and 3 were found to be significantly enriched (P<0.05) for 12, 14 and 14 Gene Ontology (GO) terms, respectively (Table I). The two most significant GO terms in module 1 were oxidation reduction (P=1.9321E-21) and metabolic process (P=1.1226E-12). The DEGs in module 1 (Fig. 3B), i.e., the cytochrome P450 (CYP) 4B1 isozyme gene (*CYP4B1*), *CYP2D10* and *CYP2D26*, which were upregulated (Table II), and *CYP2C55*, which was downregulated, were all involved in these two processes (Table I). In addition, *CYP4B1* was found to be involved in all enriched GO term functions of module 1. The terms unsaturated fatty acid, lipid, cellular lipid and fatty acid metabolic process were the most significantly enriched functions in module 2, and the upregulated gene *CYP4F14* (Fig. 3C, Table II) was predicted to be involved in all these functions (Table I). Notably, the neuropeptide signaling pathway was the most significantly enriched function (P=2.5213E-11) in module 3, and the upregulated gene tachykinin precursor 1 (*TAC1*) (Fig. 3D, Table II) was predicted to be involved in this function (Table I). The top 5 DEGs in terms of significance (Table II) were all upregulated upon microbiota depletion.

### Pathway analysis

Pathway enrichment analysis using the GenMAPP software was performed on the list of DEGs the products of which are parts of the three PPI modules. Module 1 was found to be significantly enriched for a total of 12 pathways, module 2 for 5 and module 3 for 2 (Table III). The most significant pathways in module 1 included metabolism of xenobiotics by P450s (P=2.20E-26), linoleic acid metabolism (P=6.54E-24) and arachidonic acid metabolism (P=1.49E-10). Significant pathways in module 2 also included arachidonic acid metabolism (P=5.61E-10) and linoleic acid metabolism (P=3.95E-02). Two pathways were significantly enriched in module 3, the calcium signaling pathway (P=3.23E-03) and neuroactive ligand-receptor interaction (P=5.95E-03).

## Discussion

The collective genome of the human intestinal microbiota was estimated to contain 3.3 million microbial genes, which is ~150 times more genes than the human genome. Intestinal microbiota mostly use fermentation to generate energy, converting sugars, in part, to short-chain fatty acids, which are used by the host as an energy source (1). To understand the impact of intestinal microbiota on human health, it is crucial to assess their potential function. The present study identified a total of 53 DEGs, comprising 26 upregulated and 27 downregulated genes upon depletion of the intestinal microbiota in mice. Important differences in gene expression were observed between intestinal microbiota-depleted and control mice in hierarchical clustering analysis. The PPI network of DEGs was constructed and divided into 3 modules, with the most significant module-related DEGs being *CYP4B1* in module 1, *CYP4F14* in module 2 and *TAC1* in module 3. The majority of enriched pathways of module 1 and 2 were oxidation reduction (metabolism of xenobiotics by CYPs) and lipid (e.g., linoleic and arachidonic acid) metabolism pathways. In addition, the neuropeptide signaling pathway was the most significantly enriched pathway in module 3.

Two types of functions of intestinal microbiota have been identified in a previous study, those required in all bacteria and those potentially specific to the gut (1). Functions of the first category relate to central metabolic pathways (for example, carbon metabolism and amino acid synthesis) and to important protein complexes (RNA and DNA polymerase, ATP synthase, general secretory apparatus) (1). The putative gut-specific functions include those involved in adhesion to host proteins (collagen, fibrinogen, fibronectin), or in harvesting sugars of the globo-series glycolipids, which are carried on blood and epithelial cells (1). In the present study, most of module 1-related DEGs were involved in oxidation reduction and metabolic processes such as metabolism of xenobiotics by CYPs, and the majority of module 2-related DEGs were involved in lipid metabolic processes, such as lipid metabolic process and arachidonic acid metabolism. These results suggest that the intestinal microbiota is involved in numerous metabolic and biosynthetic processes, but has particularly important roles in the regulation of lipid biosynthesis and in oxidation-reduction processes, as also indicated by previous studies (22–24).

Further analysis of the most significant DEGs *CYP4B1*, *CYP2D10*, *CYP2D26* (module 1) and *CYP4F14* (module 2) revealed that *CYP4B1* and *CYP4F14* are involved in almost all of the functions of each PPI module. In rats and rabbits, the CYP4B1 protein was shown to play an important role in mutagenic activation of procarcinogens in the organs (25). Most of organic xenobiotics require metabolic activation to electrophilic intermediates to produce adverse carcinogenic effects. Specific enzymes of the CYP superfamily are involved in the formation of reactive metabolites from certain substrates that are predicted or known occupational and environmental carcinogens (26). A new prodrug-activating enzyme system for pharmacogenic therapy of experimental brain tumors based on the rabbit CYP4B1 protein was previously described (27). CYP4Fs are a subfamily of enzymes involved in arachidonic acid metabolism and showing the highest catalytic activity towards leukotriene (LT)B4, a potent chemoattractant involved in inflammation. CYP4F-mediated metabolism of LTB4 leads to inactive ω-hydroxy products, incapable of initiating chemotaxis and the inflammatory stimuli that result in the influx of inflammatory cells (28). The *CYP4B1* and *CYP4F14* genes were identified as significantly upregulated in the present study, which, in combination with previous reports, suggests that intestinal microbiota depletion may lead to inflammation and cancer in the body.

It is notable that modules 1 and 2 were both enriched for the processes of arachidonic and linoleic acid metabolism. Arachidonic acid is a polyunsaturated ω-6 fatty acid that is released in response to tissue injury. Arachidonic acid is a pivotal signaling molecule, involved in the initiation and propagation of diverse signaling cascades regulating inflammation, pain and homeostatic functions (29). It is metabolized by three enzymatic pathways: the cyclooxygenase pathway produces prostanoid, the lipoxygenase pathway yields monohydroxy compounds and LTs, while the CYP epoxygenase pathway generates hydroxy and epoxyeicosanoids. There is increasing evidence that some of these metabolic products play critical roles in cardiovascular disease (29). Linoleic acid is predominant in dairy products and plant oils such as flax seed, and animal studies have reported a reduction in intra-abdominal fat and an enhanced gain in fat-free mass upon linoleic acid supplementation; another study reported linoleic acid-mediated whole-body fat loss in overweight men and women; there have also been some concerns that linoleic acid can promote oxidative stress and induce hepatic lipid accumulation (30–32). Based on these studies and the present findings on arachidonic and linoleic acid metabolism, the two processes appear to play a key role in human health and to be closely linked to the balance of intestinal microbiota.

In contrast to the reported effects of intestinal microbiota on oxidation reduction and lipid metabolism (30–32), an association between intestinal microbiota and the neuropeptide signaling pathway has not been previously reported. In our study, it is notable that the neuropeptide signaling pathway was the most significantly enriched pathway in module 3. Among the here-identified DEGs, *TAC1* is predicted to be involved in this pathway. This gene encodes a neurotransmitter of the central and peripheral nervous system (33), and the protein has additionally been associated with immunologic and inflammatory processes (34). The gut and the brain are closely connected organs, and their interaction plays an important role not only in gastrointestinal function, but also in certain feeling states and in intuitive decision making (35); alterations in this interaction have been associated with a wide range of disorders, including functional, inflammatory gastrointestinal, and eating disorders. It has been reported that healthy humans and rats produce autoantibodies directed against appetite-regulating peptide hormones and neuropeptides, suggesting that these autoantibodies may play physiological roles in hunger- and satiety-related pathways (36). Gut-related antigens including those produced by the intestinal microflora, may affect the production of these autoantibodies, which might represent a new link between the gut and the regulation of appetite. We thus argue that the depletion of the intestinal microflora in mice may lead to impaired neuropeptide signaling.

In conclusion, our findings strongly suggest that intestinal microbiota depletion affects metabolism, oxidation reduction and neuropeptide signaling pathways in mice, involving a number of genes and interactions. Numerous diseases, as well as aging, can be induced by depletion of the intestinal microflora, and therefore, the dynamic equilibrium of the intestinal microflora plays a key role in human health. The neuropeptide signaling pathway was first reported in the present study to be affected by the depletion of the intestinal microflora, a result which reveals a potential link between the intestinal bacteria and the nervous system. However, further experimentation and additional studies are needed to confirm this link; such studies are expected to enhance our understanding of the interactions between the bacteria of the intestinal microflora and their host environment.

## Figures and Tables

**Table I:** Functional annotation of the genes in the three modules using Gene Ontology (GO) terms.

A, Significantly enriched GO terms (n=12) and associated DEGs in module 1			

GO id	Corr. P	Genes in test set	Functional description
55114	1.9321E-21	*CYP2J5, CYP2C70, CYP2D9, CYP2D10, CYP2C37, CYP2C55, CYP3A25, CYP2C54, CYP3A13, CYP2C44, CYP3A11, CYP2C29, CYP2C40, CYP2E1, CYP1A2, CYP4B1, CYP2D26*	Oxidation reduction
8152	1.1226E-12	*CYP2J5, CYP2C70, CYP2D9, CYP2D10, CYP2C37, CYP2C55, CYP3A25, CYP2C54, CYP3A13, CYP2C44, CYP3A11, CYP2C29, UGT2B1, CYP2C40, CYP2E1, CYP1A2, UGT1A1, UGT3A2, CYP4B1, CYP2A12, UGT2B35, UGT2B5, CYP2D26*	Metabolic process
42537	9.6276E-10	*UGT2B1, CYP1A2, UGT1A1, CYP4B1*	Benzene and derivative metabolic process
6805	2.6493E-08	*UGT2B1, CYP1A2, UGT1A1, CYP4B1*	Xenobiotic metabolic process
71466	2.6493E-08	*UGT2B1, CYP1A2, UGT1A1, CYP4B1*	Cellular response to xenobiotic stimulus
6725	2.6493E-08	*CYP2A12, UGT2B35, UGT2B1, CYP1A2, UGT1A1, CYP4B1*	Cellular aromatic compound metabolic process
9410	3.7437E-08	*UGT2B1, CYP1A2, UGT1A1, CYP4B1*	Response to xenobiotic stimulus
70887	4.26E-04	*UGT2B1, CYP1A2, UGT1A1, CYP4B1*	Cellular response to chemical stimulus
44248	1.01E-03	*UGT2B35, UGT2B1, CYP1A2, UGT1A1, CYP4B1*	Cellular catabolic process
9056	3.27E-03	*UGT2B35, UGT2B1, CYP1A2, UGT1A1, CYP4B1*	Catabolic process
42221	7.25E-03	*UGT2B1, CYP2D26, CYP1A2, UGT1A1, CYP4B1*	Response to chemical stimulus
51716	9.66E-03	*UGT2B1, CYP1A2, UGT1A1, CYP4B1*	Cellular response to stimulus

B, Significantly enriched GO terms (n=14) and associated genes in module 2			

GO id	Corr. P	Genes in test set	Functional description

33559	2.0285E-08	*PTGS2, PTGS1, CYP4F14, ALOX5*	Unsaturated fatty acid metabolic process
6629	1.1496E-07	*PTGS2, PLB1, PTGS1, PLA2G2A, CYP4F14, ALOX5*	Lipid metabolic process
44255	1.2555E-06	*PTGS2, PTGS1, PLA2G2A, CYP4F14, ALOX5*	Cellular lipid metabolic process
6631	2.1203E-06	*PTGS2, PTGS1, CYP4F14, ALOX5*	Fatty acid metabolic process
32787	9.1722E-06	*PTGS2, PTGS1, CYP4F14, ALOX5*	Monocarboxylic acid metabolic process
43436	5.05E-05	*PTGS2, PTGS1, CYP4F14, ALOX5*	Oxoacid metabolic process
19752	5.05E-05	*PTGS2, PTGS1, CYP4F14, ALOX5*	Carboxylic acid metabolic process
6082	5.05E-05	*PTGS2, PTGS1, CYP4F14, ALOX5*	Organic acid metabolic process
42180	5.26E-05	*PTGS2, PTGS1, CYP4F14, ALOX5*	Cellular ketone metabolic process
55114	8.20E-05	*PTGS2, PTGS1, CYP4F14, ALOX5*	Oxidation reduction
44281	1.02E-03	*PTGS2, PTGS1, CYP4F14, ALOX5*	Small molecule metabolic process
44238	1.89E-03	*PTGS2, PLB1, PTGS1, PLA2G2A, CYP4F14, ALOX5*	Primary metabolic process
8152	3.03E-03	*PTGS2, PLB1, PTGS1, PLA2G2A, CYP4F14, ALOX5*	Metabolic process
44237	9.30E-03	*PTGS2, PTGS1, PLA2G2A, CYP4F14, ALOX5*	Cellular metabolic process

C, Significantly enriched GO terms (n=14) and associated genes in module 3			

GO id	Corr. P	Genes in test set	Functional description

7218	2.5213E-11	*GRP, TACR3, TACR2, TACR1, TAC1, TAC2*	Neuropeptide signaling pathway
8015	1.8064E-10	*NTS, TACR3, TACR1, AGT, TAC1, TAC2*	Blood circulation
3013	1.8064E-10	*NTS, TACR3, TACR1, AGT, TAC1, TAC2*	Circulatory system process
7186	1.1712E-07	*GRP, TACR3, TACR2, TACR1, AGT, TAC1, GAST, TAC2*	G-protein coupled receptor protein signaling pathway
7166	8.8661E-07	*GRP, TACR3, TACR2, TACR1, AGT, TAC1, GAST, TAC2*	Cell surface receptor linked signaling pathway
23033	4.6021E-06	*GRP, TACR3, TACR2, TACR1, AGT, TAC1, GAST, TAC2*	Signaling pathway
51239	4.8121E-06	*GRP, TACR3, TACR2, TACR1, AGT, TAC1*	Regulation of multicellular organismal process
3008	4.9678E-06	*NTS, TACR3, TACR2, TACR1, AGT, TAC1, TAC2*	System process
65008	1.24E-05	*NTS, TACR3, TACR1, AGT, TAC1, TAC2*	Regulation of biological quality
23052	1.33E-05	*GRP, TACR3, TACR2, TACR1, AGT, TAC1, GAST, TAC2*	Signaling
65007	4.55E-05	*GRP, NTS, TACR3, TACR2, TACR1, AGT, TAC1, GAST, TAC2*	Biological regulation
32501	4.32E-04	*NTS, TACR3, TACR2, TACR1, AGT, TAC1, TAC2*	Multicellular organismal process
50794	2.08E-03	*GRP, TACR3, TACR2, TACR1, AGT, TAC1, GAST*	Regulation of cellular process
50789	2.69E-03	*GRP, TACR3, TACR2, TACR1, AGT, TAC1, GAST*	Regulation of biological process

Corr. P, corrected p-value; *CYP*, cytochrome P450 gene; *TAC1*, tachykinin precursor 1 gene.

**Table II:** Characteristics of the most significant differentially expressed genes in the 3 modules.

Id	Gene symbol	FDR	|log FC|	GO terms^a^	Regulation
ILMN_2790496	*CYP4B1*	0.0023580	2.64	All in Table IA	Up
ILMN_1229535	*CYP2D10*	0.0234023	1.42	55114, 8152	Up
ILMN_2704777	*CYP2D26*	0.0190232	1.92	55114, 8152, 42221	Up
ILMN_1231625	*CYP4F14*	0.0055720	1.31	All in Table IB	Up
ILMN_1251000	*TAC1*	0.0257095	1.09	All in Table IC	Up

a The numbers denote Gene Ontology (GO) ids shown in Table I.

FC, fold change; CYP, cytochrome P450; TAC1, tachykinin precursor 1; FDR, false discovery rate.

**Table III:** Pathway enrichment analysis of differentially expressed genes in the three modules based on information from the Kyoto Encyclopedia of Genes and Genomes (KEGG) pathways database for *Mus musculus* (mmu).

Module	KEGG id	P
1	mmu00980: Metabolism of xenobiotics by cytochrome P450	2.20E-26
	mmu00830: Retinol metabolism	3.63E-26
	mmu00591: Linoleic acid metabolism	6.54E-24
	mmu00983: Drug metabolism	1.30E-10
	mmu00590: Arachidonic acid metabolism	1.49E-10
	mmu00140: Steroid hormone biosynthesis	5.88E-09
	mmu00053: Ascorbate and aldarate metabolism	1.18E-03
	mmu00040: Pentose and glucuronate interconversions	1.52E-03
	mmu00860: Porphyrin and chlorophyll metabolism	4.74E-03
	mmu00150: Androgen and estrogen metabolism	4.74E-03
	mmu00500: Starch and sucrose metabolism	6.77E-03
	mmu00232: Caffeine metabolism	3.10E-02
2	mmu00590: Arachidonic acid metabolism	5.61E-10
	mmu04370: Vascular endothelial growth factor signaling pathway	1.69E-03
	mmu00592: α-linolenic acid metabolism	1.56E-02
	mmu00565: Ether lipid metabolism	3.01E-02
	mmu00591: Linoleic acid metabolism	3.95E-02
3	mmu04020: Calcium signaling pathway	3.23E-03
	mmu04080: Neuroactive ligand-receptor interaction	5.95E-03
